# Photoinduced Water Oxidation in Chitosan Nanostructures Containing Covalently Linked Ru^II^ Chromophores and Encapsulated Iridium Oxide Nanoparticles

**DOI:** 10.1002/chem.202102032

**Published:** 2021-09-23

**Authors:** Giuseppina La Ganga, Fausto Puntoriero, Enza Fazio, Mirco Natali, Francesco Nastasi, Antonio Santoro, Maurilio Galletta, Sebastiano Campagna

**Affiliations:** ^1^ Dipartimento di Scienze Chimiche Biologiche Farmaceutiche ed Ambientali Università di Messina 98166 Messina Italy; ^2^ Dipartimento di Scienze Matematiche e Informatiche Scienze Fisiche e Scienze della Terra Università di Messina 98166 Messina Italy; ^3^ Dipartimento di Scienze Chimiche Farmaceutiche ed Agrarie Università di Ferrara 44121 Ferrara Italy

**Keywords:** artificial photosynthesis, electron transfer, photochemical water oxidation, photochemistry; ruthenium

## Abstract

The luminophore Ru(bpy)_2_(dcbpy)^2+^ (bpy=2,2’‐bipyridine; dcbpy=4,4’‐dicarboxy‐2,2’‐bipyridine) is covalently linked to a chitosan polymer; crosslinking by tripolyphosphate produced Ru‐decorated chitosan fibers (NS‐RuCh), with a 20 : 1 ratio between chitosan repeating units and Ru^II^ chromophores. The properties of the Ru^II^ compound are unperturbed by the chitosan structure, with NS‐RuCh exhibiting the typical metal‐to‐ligand charge‐transfer (MLCT) absorption and emission bands of Ru^II^ complexes. When crosslinks are made in the presence of IrO_2_ nanoparticles, such species are encapsulated within the nanofibers, thus generating the IrO_2_⊂NS‐RuCh system, in which both Ru^II^ photosensitizers and IrO_2_ water oxidation catalysts are within the nanofiber structures. NS‐RuCh and IrO_2_⊂NS‐RuCh have been characterized by dynamic light scattering, scanning electronic microscopy, and energy‐dispersive X‐ray analysis, which indicated a 2 : 1 ratio between Ru^II^ chromophores and IrO_2_ species. Photochemical water oxidation has been investigated by using IrO_2_⊂NS‐RuCh as the chromophore/catalyst assembly and persulfate anions as the sacrificial species: photochemical water oxidation yields O_2_ with a quantum yield (*Φ*) of 0.21, definitely higher than the *Φ* obtained with a similar solution containing separated Ru(bpy)_3_^2+^ and IrO_2_ nanoparticles (0.05) or with respect to that obtained when using NS‐RuCh and “free” IrO_2_ nanoparticles (0.10). A fast hole‐scavenging process (rate constant, 7×10^4^ s^−1^) involving the oxidized photosensitizer and the IrO_2_ catalyst within the IrO_2_⊂NS‐RuCh system is behind the improved photochemical quantum yield of IrO_2_⊂NS‐RuCh.

## Graphical abstract

**Restricted environment frees up reaction**: Chitosan fibers with covalently linked Ru^II^ polypyridine complexes and encapsulated IrO_2_ nanoparticles exhibit a remarkable quantum yield of 0.21 for photoinduced water oxidation in sacrificial schemes. The restricted environment is held responsible for accelerating electron transfer steps, which are considered to be the basis of the efficiency of the overall photocatalytic process.

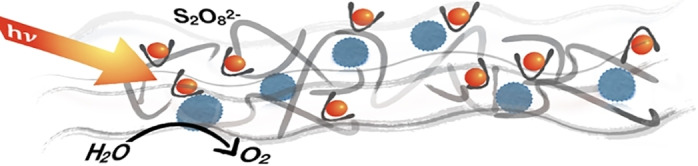

## Introduction

The development of artificial photosynthetic systems, that is synthetic systems capable of efficiently converting light energy into chemical energy inspired by the photosynthetic process performed by natural organisms, is attracting a large interest nowadays, for both fundamental and applicative reasons, in particular because of the potential impact on renewable energy research.^[1]^ Within this general frame, the development of new nanomaterials that integrate all necessary components (i. e., light‐harvesting, charge separation and catalyst subunits) into a restricted environment is a promising research field,^[2]^ as a restricted environment can speed up the rate (and efficiency) of photoinduced electron transfer processes, when bimolecular reactions are involved.

**Figure 1:**
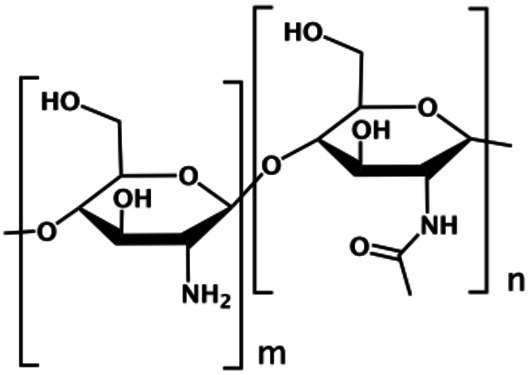
Representation of a chitosan repetitive unit.

In this work, an abundant natural polymer, chitosan, is exploited as a useful environment to integrate light harvesting systems‐which can also play the role of initiator of the photoinduced charge separation process, that is the charge separation unit—and catalysts in order to achieve photoinduced water oxidation, considered one of the bottlenecks of water splitting. Chitosan is a well‐known amino‐polysaccharide (Figure 1) characterized by interesting biological and chemical properties, including biocompatibility, biodegradability, non‐toxicity, physiological inertness and antibacterial properties.^[3]^ Whereas most of these properties are not relevant to our scopes, we planned to take advantage of the supramolecular structure of chitosan, in particular of its ability to incorporate small molecules into its fiber‐like nanostructures.^[4]^ Indeed, the polymeric structure of chitosan, in the presence of various agents, like tripolyphosphate (TPP), can rearrange to form nanosized superstructures that can encapsulate several substrates.^[4]^

Herein, we describe the synthesis and characterization of hybrid nanosystems made of chitosan nanostructures decorated with Ru^II^ polypyridine complexes and loaded with iridium oxide nanoparticles as well‐known water oxidation catalyst.[^5^, ^6^] The photophysical properties and the photocatalytic performances of these hybrid nano‐systems as far as photoinduced water oxidation is concerned are presented and discussed.

## Results and Discussion

### Synthesis of chitosan containing covalently linked Ru(bpy)_3_^2+^‐type subunits (polymeric RuCh)

In order to prepare nanostructures of chitosan decorated with Ru^II^ polypyridine complexes, it is necessary to find a synthetic way to covalently link Ru^II^ complexes to the chitosan structure via a NH bond. This necessity arises from the fact that positively charged species, such as Ru(bpy)_3_^2+^ (bpy=2,2’‐bipyridine), are not easily incorporated into chitosan nanostructures because of the positive nature of the chitosan structure itself; in any case, the non covalently linked sensitizer possibly incorporated into the chitosan structure would easily be released in water. In fact, this is what happened in preliminary experiments (not reported) of this study. This prompted us to functionalize the chitosan basic molecules to allow the incorporation of Ru(bpy)_3_^2+^‐like species by direct link to the chitosan structure.

**Scheme 1:**
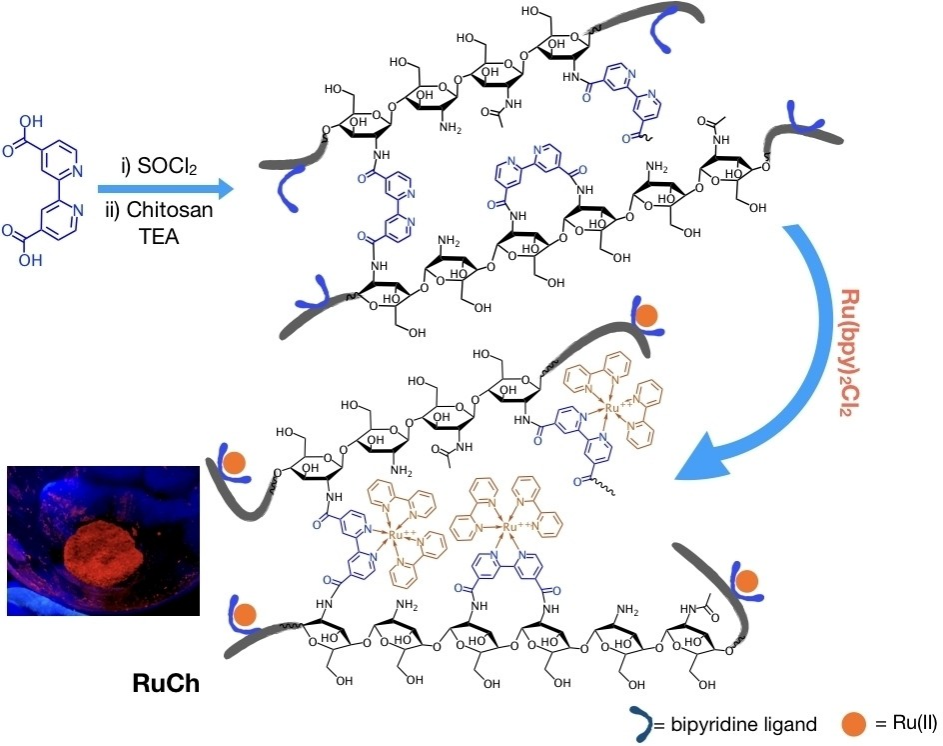
Schematic representation of the synthesis of RuCh. Inset: Photo of RuCh powder under UV light.

The synthetic route to obtain a ruthenium polypyridine complex linked to the chitosan structure (RuCh**)**, is described in Scheme 1.

In the first step of the synthetic scheme, 4,4’‐dicarboxy‐2,2’‐bipyridine (dcbpy) is transformed in the corresponding acyl chloride, in order to easily react with the ammino group of chitosan. The so‐formed bpyNHCH is then reacted with the ruthenium complex [Rubpy_2_Cl_2_] (bpy=2,2’‐bipyridine) to obtain a modified chitosan containing covalently linked {Ru(bpy)_3_}^2+^ subunits, RuCh.

**Figure 2:**
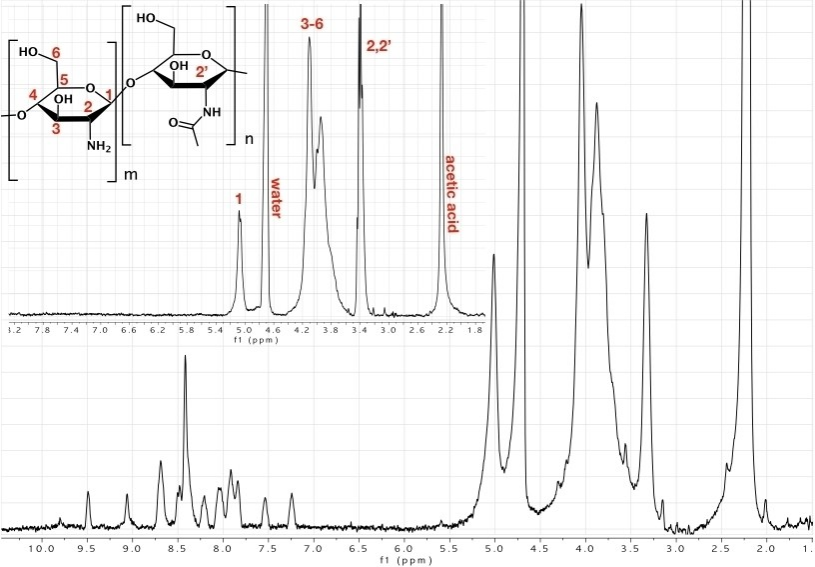
^1^H NMR spectrum of RuCh in D_2_O (CD_3_COOD, 1 % *v*/*v*). The ^1^H NMR spectrum of low‐molecular‐weight chitosan in D_2_O (CD_3_COOD, 1 % *v*/*v*) is shown in the inset. Spectra were recorded at 40 °C.

We characterized RuCh by H NMR in acidic conditions, at 40 °C, because of its low solubility; its H NMR spectrum, in comparison with the H NMR spectrum of “free” chitosan (i. e., the chitosan missing the bpy functionalization and the ruthenium complex), is reported in Figure 2.

As shown in Figure 2, the various peaks in the range 5–1.8 ppm can be assigned to the chitosan structure and the peaks in the range 10–7 ppm are attributed to the bpy ligand of the ruthenium complex linked to chitosan. Moreover, from the NMR data it is also possible to establish a chitosan/Ru ratio of 20 : 1 (Figure S1 in the Supporting Information) so it can be stated that, on average, one ruthenium complex is linked per 20 units of chitosan monomer, by considering the monomer as reported in Figure 2.

### Nanosized superstructures prepared from RuCh (NS‐RuCh and IrO_2_⊂NS‐RuCh)

**Scheme 2:**
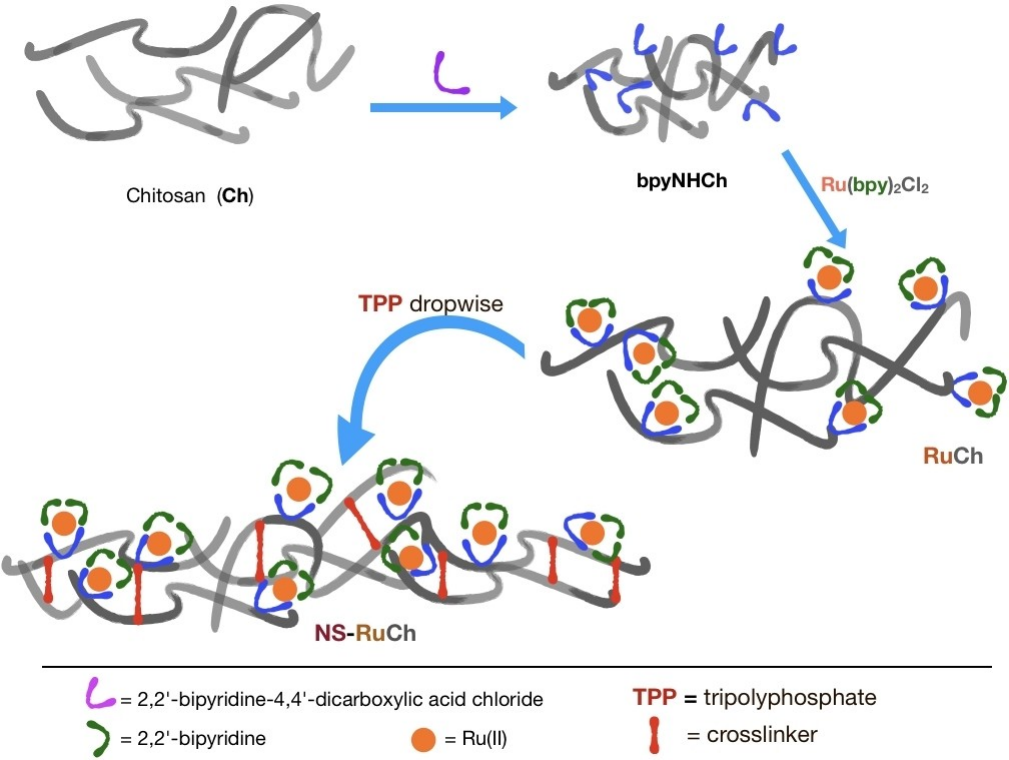
A cartoon representation of NS‐RuCh synthesis by using the RuCh polymer.

We prepared nanosized superstructures starting from RuCh by using a modification of methods already known in literature,^[4]^ taking advantage of the fact that RuCh maintains the same reactivity of the free chitosan. In particular, we used TPP as the crosslinker, so obtaining nanoparticles containing the {Ru(bpy)_3_}^2+^ subunits (NS‐RuCh). The synthetic method is schematized in Scheme 2.

**Figure 3:**
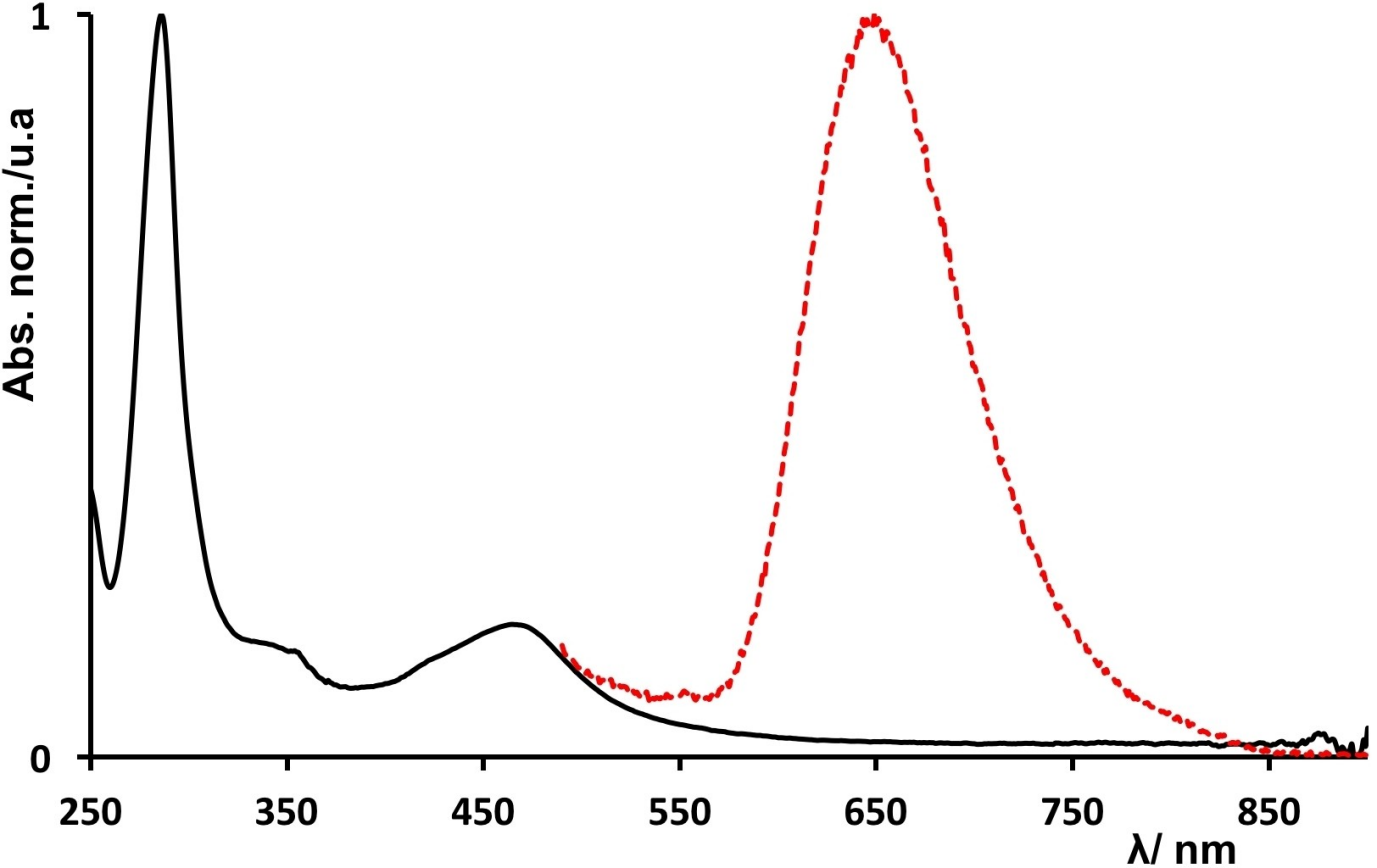
Absorption and emission spectra of NS‐RuCh in phosphate buffer (20 mM, pH 7).

The absorption and emission spectra of NS‐RuCh in phosphate buffer are dominated by the spectra of its Ru(bpy)_3_^2+^‐type subunits (Figure 3): the UV region is characterized by the bpy‐centered band at around 280 nm and the visible region exhibits the typical metal‐to‐ligand charge‐transfer (MLCT) band of Ru^II^ polypyridine complexes.[^7^, ^8^] The emission is attributed to the ^3^MLCT excited state of the Ru(bpy)_3_^2+^‐type subunits. Both the maxima of the visible absorption spectrum (465 nm) and of the emission band (645 nm) are slightly red‐shifted compared to those of the model Ru(bpy)_3_^2+^ species (e. g., the emission spectrum of the model species exhibits a maximum at 625 nm in the identical experimental condition^[7]^), as expected since the acceptor of the MLCT transition in the present case is the modified bpy ligand, which has a lower lying π* orbital than unsubstituted bpy. The emission lifetime in phosphate buffer at pH 7 is 305 ns, also in agreement with the ^3^MLCT assignment. The spectroscopic and excited state data indicate that the Ru(bpy)_3_^2+^‐type chromophores maintain their characteristic photophysical properties in the NS‐RuCh assemblies.

Once verified that the properties of the Ru‐based chromophores are maintained in the NS‐RuCh assemblies, we performed the preparation of crosslinked Ru‐decorated chitosan nanostructures in the presence of IrO_2_ nanoparticles in solution, which are known to be efficient water oxidation catalysts.[^9^, ^10^, ^11^] The assemblies so prepared contain both Ru(bpy)_3_^2+^‐type chromophores, covalently linked to the chitosan structure, and IrO_2_ nanoparticles, encapsulated within the superstructure of NS‐RuCh (i. e., a IrO_2_⊂NS‐RuCh system), as demonstrated by dynamic light scattering (DLS), scanning electronic microscopy (SEM), and energy‐dispersive X‐ray analysis (EDX).

**Figure 4:**
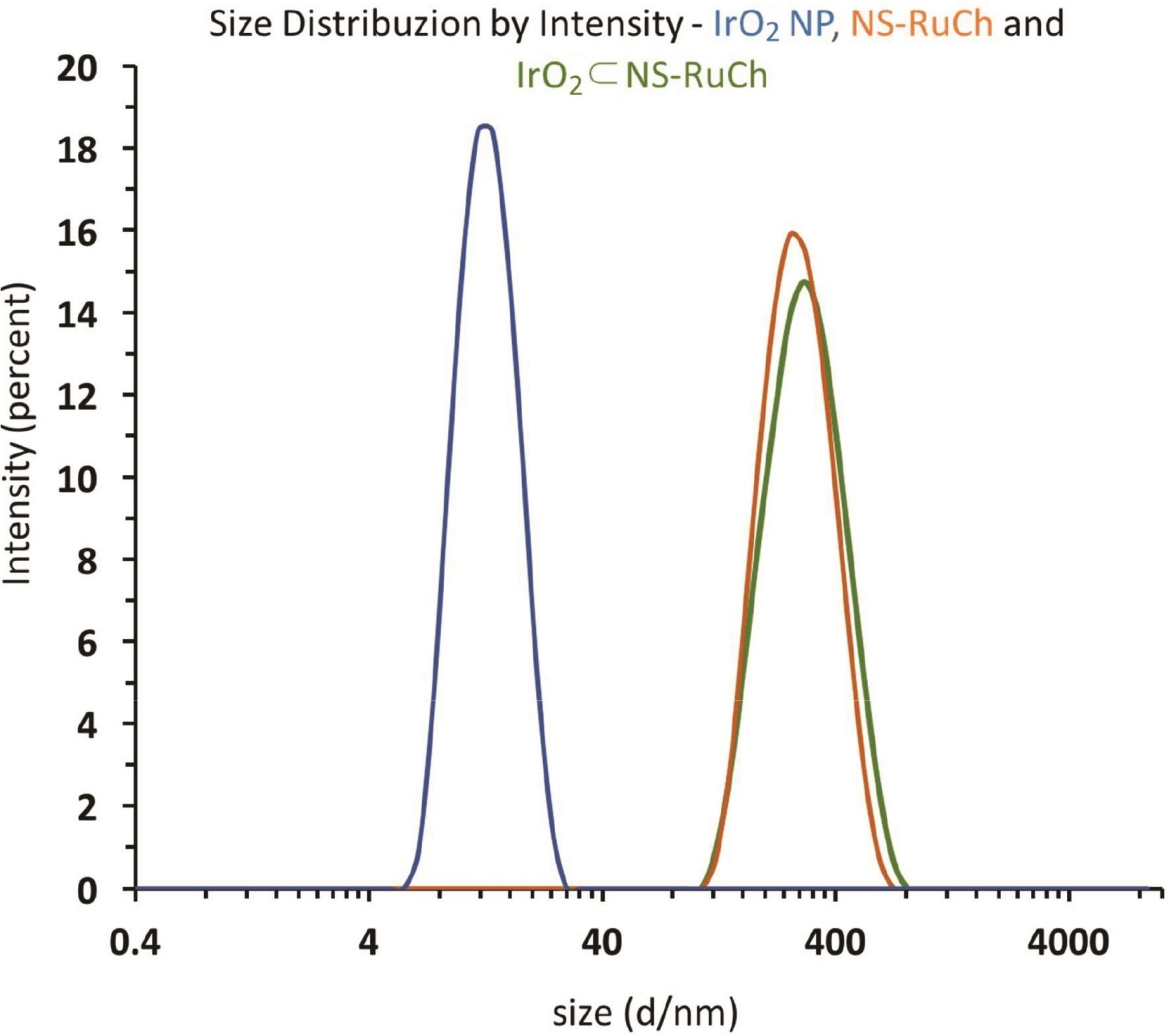
DLS analysis of IrO_2_ nanoparticles (blue), NS‐RuCh (orange) and IrO_2_⊂NS‐RuCh (green). Note that the *x*‐axis is drawn on a logarithmic scale.

**Figure 5:**
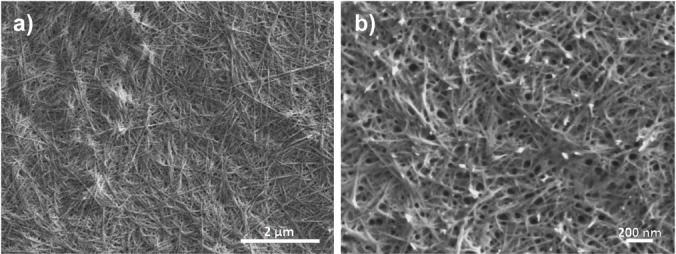
SEM analysis of IrO_2_⊂NS−Ru at different scales.

Dynamic light scattering analysis revealed an average size of 340 nm for IrO_2_⊂NS−Ru, significantly larger than that observed in the case of NS‐RuCh missing the IrO_2_ nanoparticle, that was 250 nm (Figure 4). The SEM images shows (Figure 5) a three dimensional porous net with a smooth surface. The nanofibers have an average diameter of about 250 nm and are spatially dispersed in random orientations. EDX analysis of such structures revealed the presence of both Ru and Ir atoms and allowed to estimate a Ru/Ir ratio of 2 : 1 in IrO_2_⊂NS‐RuCh, confirming the efficient encapsulation of IrO_2_ nanoparticles in the assemblies (Figure S2). Interestingly, once synthetically prepared in IrO_2_⊂NS‐RuCh, IrO_2_ nanoparticles are not released in solution, as clearly demonstrated by the absence of any traces of “free” IrO_2_ nanoparticles in the DLS experiments on IrO_2_⊂NS‐RuCh. Probably, the several hydroxy groups which are present in the chitosan structure further stabilize the IrO_2_ nanoparticles within the structure.

**Figure 6:**
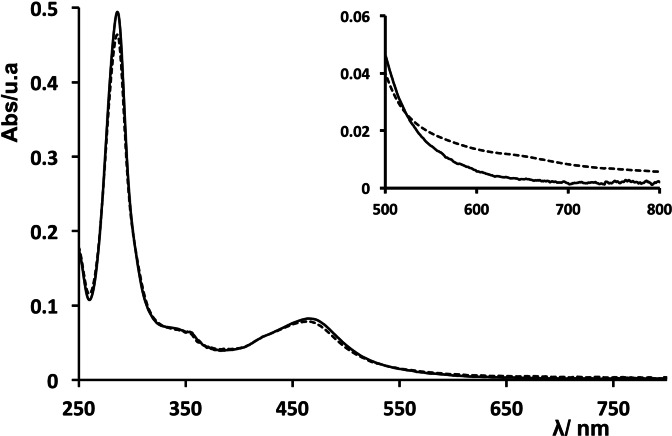
Absorption spectra of NS‐RuCh (solid line) and IrO_2_⊂NS‐RuCh (dashed line) in phosphate buffer (20 mM, pH 7). The inset shows a zoomed area (500–800 nm) of the spectra.

The absorption spectrum of IrO_2_⊂NS‐RuCh in phosphate buffer at pH 7 strongly overlaps with that of NS‐RuCh (Figure 6), with the remarkable difference that the spectrum of IrO_2_⊂NS‐RuCh shows a small additional contribution at about 650 nm which can be attributed to the presence of IrO_2_ nanoparticles.[^9^, ^10^, ^11^, ^12^]

The emission spectrum of the IrO_2_⊂NS‐RuCh is identical to that of NS‐RuCh, and the emission lifetime is also very similar (315 ns): altogether, these data indicate that there is no significant interactions between the Ru‐based chromophore and the IrO_2_ catalyst, which thus behave as independent entities.

### Photo‐induced water oxidation

Photoinduced water oxidation using Ru^II^ polypyridine complexes has been explored by a catalytic cycle involving IrO_2_ nanoparticles and sacrificial donor agents in solution.[^9^, ^10^, ^11^] Sacrificial agents have the role of extracting an electron from the MLCT excited state, simulating the effect of semiconductors, like nanostructured TiO_2_, in regenerative cells.^[13]^ Whereas regenerative cells, for example dye‐sensitized photosynthetic cells (DSPSC),^[13b]^ are needed for effective water splitting, the use of sacrificial agents in solution allows to investigate the rate constants of the individual processes involved.

Sodium persulfate is a quite convenient sacrificial agent, since it efficiently quenches the MLCT state of Ru^II^ polypyridine complexes by oxidative electron transfer, leading to the oxidized form of the chromophore, which is able to oxidize a suitable multielectron transfer catalyst like IrO_2_ nanoparticles.^[9]^ The overall reaction process is summarized in Equations (1)–(3),^[14]^ where Ru^2+^ represents the Ru^II^ polypyridine chromophore, SA is the sacrificial agent (sodium persulfate in this case), and C is the catalyst (IrO_2_ nanoparticles). Moreover, the reduced form of persulfate (i. e., the reduced SA species in Equation (2)) is not stable, forming sulfate radical anion, which is even a better oxidant than persulfate and reacts with Ru^II^ polypyridine complexes so contributing to produce the oxidized photosensitizer Ru^3+^. Therefore, in sacrificial schemes involving persulfate anions, two Ru^3+^ equivalent species are produced by a single absorbed photon.

(1) $$\mathrm{Ru}{}^{2+}+h\nu \to {}^{*}\mathrm{Ru}{}^{2+}$$

(2) $${}^{*}\mathrm{Ru}{}^{2+}+\mathrm{SA}\to \mathrm{Ru}{}^{3+}+\mathrm{reduced}\;\mathrm{SA}\;\mathrm{decomposition}\;\mathrm{products}$$

(3) $$\mathrm{Ru}{}^{3+}+C\to \mathrm{Ru}{}^{2+}+C{}^{+}$$

The reaction in Equation (3) is repeated, involving several oxidized forms of C, in a stepwise manner, represented by Equation (4), until the active catalytic species is formed – for example C^4+^ – and finally water oxidation takes place, according to Equation 5.

(4) $$\mathrm{Ru}{}^{3+}+C{}^{(n-1)+}\to \mathrm{Ru}{}^{2+}+C{}^{n+}$$

(5) $$C{}^{4+}+2\;H{}_{2}O\to C+O{}_{2}+4H{}^{+}$$

Clearly, several poisoning reactions can take place, reducing the quantum yield of the overall process. Such poisoning processes include direct decay of ^*^Ru^2+^ to the ground state, which competes with Equation (2), as well as any back electron transfer process, which anyway is minimized – at least for the back electron transfer involving SA – by the instability of the reduced form of persulfate anions. Please note that since persulfate anions can produce two Ru^III^ polypyridine complexes per absorbed photons (see above), and probably C^4+^ is needed to oxidize water, the maximum photochemical water oxidation quantum yield in systems involving Ru^II^ polypyridine complexes, persulfate anions as SA and IrO_2_ nanoparticles as catalyst is 0.5.

**Figure 7:**
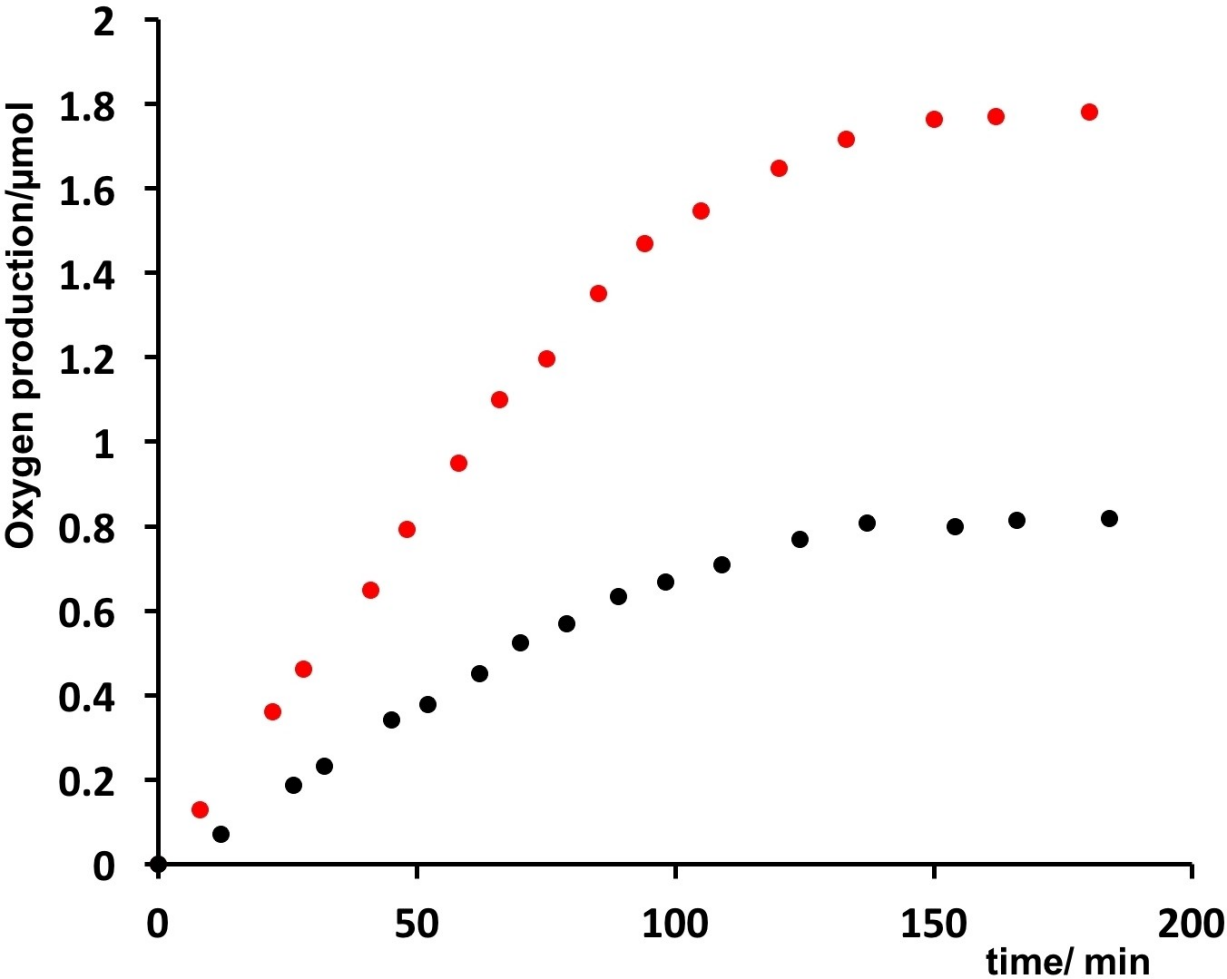
Oxygen evolution in the IrO_2_⊂NS−Ru/Na_2_S_2_O_8_ (red) and NS‐RuCh/IrO_2_/Na_2_S_2_O_8_ systems (black).

We studied the photoproduction of molecular oxygen by irradiating (*λ*=450 nm) 2 mL of a buffered solution (20 mM phosphate buffer at pH 7) of IrO_2_⊂NS−Ru (1×10^−4^ M) and Na_2_S_2_O_8_ (10 mM). The concentration of IrO_2_⊂NS−Ru refers to the concentration of Ru(bpy)_3_^2+^‐type subunit, calculated on the basis of the molar absorption coefficient of RuCh (assumed roughly identical to that of Ru(bpy)_3_^2+^ is aqueous solution, i. e., 14 600 M^−1^ cm^−1^ ^[7b]^). The amount of molecular oxygen evolved during the photocatalytic cycle as well as the quantum yield of the process have been determined as previously described.^[11]^ A typical result is shown in Figure 7. Similar experiments have been made by using Ru(bpy)_3_^2+^ or NS‐RuCh as photosensitizers and adding IrO_2_ nanoparticles in solution, always in the presence of persulfate, for comparison purposes. The three set of systems are schematized as follows:

- Ru(bpy)_3_Cl_2_/IrO_2_/Na_2_S_2_O_8_. This system is very well known in literature and is here used as a benchmark.[^9^, ^10^, ^11^]
- NS‐RuCh/IrO_2_/Na_2_S_2_O_8_. In this system, the IrO_2_ catalyst is added in solution to the preformed NS‐RuCh assemblies containing the photosensitizer.
- IrO_2_⊂NS‐RuCh/Na_2_S_2_O_8_. In this system the IrO_2_ catalyst is encapsulated within the nanostructured assemblies.

In all the experiments, the concentration of the photosensitizer and catalyst are chosen to be constant (1×10^−4^ and 5×10^−5^ M, respectively, to respect the 2 : 1 photosensitizer/catalyst ratio estimated in IrO_2_⊂NS‐RuCh. Please note that concentration of IrO_2_ catalyst is referred to iridium atom content). Table 1 collects the photochemical quantum yields of molecular oxygen production recorded for the systems (a)–(c).

**Table 1:** Quantum yield of oxygen production measured for each system reported.^[a]^

System	*Φ*
a) Ru(bpy)_3_Cl_2_ /IrO_2_/ Na_2_S_2_O_8_	0.05
b) NS‐RuCh/ IrO_2_/Na_2_S_2_O_8_	0.10
c) IrO_2_⊂NS‐RuCh**/**Na_2_S_2_O_8_	0.21^[b]^

[a] Each value is an average of three independent experiments in which a solution of 2 mL in buffer phosphate at pH 7 containing the photosensitizer (1×10^−4^ M), the catalyst (5×10^−5^ M) and Na_2_S_2_O_8_ (10 mM) was irradiated at *λ*=450 nm. [b] In this system the concentration of the catalyst is estimated to be 5x10^−5^ M by the determination of 2 : 1 Ru/Ir ratio obtained by EDX experiments (see text).

The larger photochemical quantum yield of molecular oxygen production in system (b) compared to that of system (a) can be justified by considering that an IrO_2_ nanoparticle which has been involved in one of the electron‐transfer steps of Equations (3) and (4), so generating an intermediately oxidized form of IrO_2_, is probably closer to other photosensitizers, so experiencing a higher local concentration of photosensitizers, making the overall photochemical process more efficient.

System (c), in which Ru(bpy)_3_^2+^‐type chromophores are covalently linked to the chitosan nanofibers and IrO_2_ nanoparticles are encapsulated within the nanostructure by the synthetic approach, experiences an even higher local concentration of both photosensitizers and catalyst: therefore it is not surprising that the photochemical quantum yield of molecular oxygen obtained using IrO_2_⊂NS−Ru is larger than the other systems (a) and (b). The improved efficiency of the photocatalytic process in the case of IrO_2_⊂NS−Ru can be thus attributed to kinetic advantages favored by the proximity of sensitizer and catalyst. This attribution is confirmed by flash photolysis experiments (see below).

It can be noted that the photostability of the systems based on Ru^II^ polypyridine complexes as photosensitizers is limited by the stability of the oxidized form of the photosensitizer, which can undergo oxidation of the polypyridine ligands.^**[15]**^ This is evidenced by modification of the absorption spectrum of the photosensitizer during the photocatalysis. Noteworthy, system (c) exhibits a significant higher stability than system (a), probably connected with the faster reaction involving the oxidized form of the sensitizer (Figures S3–S5).

### Flash photolysis experiments

**Figure 8:**
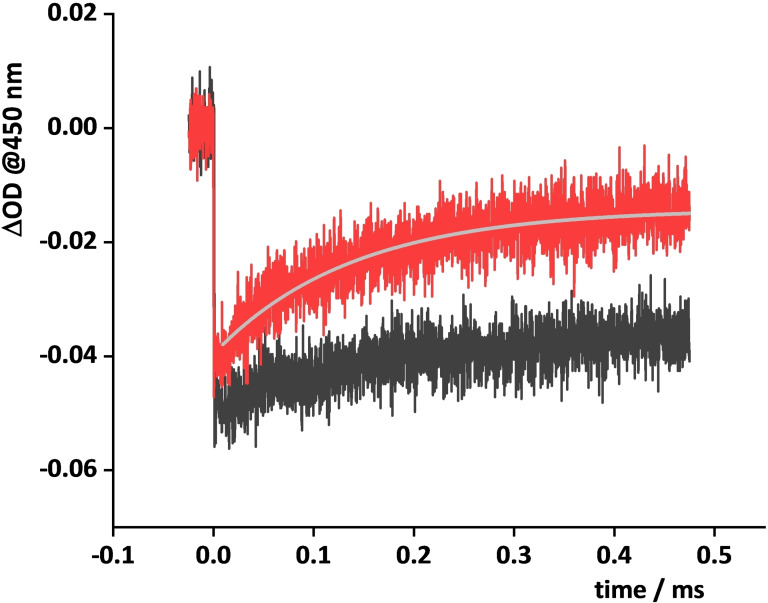
Flash photolysis experiments: *λ*_ex_=355 nm; Na_2_S_2_O_8_ (1×10^−2^ M). IrO_2_⊂NS−Ru (red) and NS‐RuCh/IrO_2_ (5×10^−5^) systems (black).

In order to better understand the photocatalytic performance of the IrO_2_⊂NS−Ru system, we performed flash photolysis experiments in presence of Na_2_S_2_O_8_. These experiments allow to gain kinetic information on the hole scavenging reaction between the oxidized photosentitizer and the catalyst, that is the key reaction in Equation (3).[^9^, ^10^, ^13^, ^16^, ^17^, ^18^, ^19^] Figure 8 shows the kinetics of the bleaching and recovery of the MLCT absorption at 450 nm for the IrO_2_⊂NS‐RuCh system and for the NS‐RuCh in the presence of 5×10^−5^ M of “free” IrO_2_, which testifies the hole scavenging process. The bleaching of the MLCT band is produced upon excitation of the Ru‐based chromophore followed by very fast oxidative electron transfer by 0.01 M persulfate anions, so generating the Ru^III^ species, which may undergo hole scavenging by the IrO_2_ catalyst, synthetically incorporated in IrO_2_⊂NS‐RuCh or present in solution in the case of NS‐RuCh. The overall concentration of IrO_2_, either incorporated or in solution, is identical for comparison purposes (5 x 10^−5^ M). It is clear from Figure 8 that the hole scavenging process (associated to the bleach recovery due to the restoring of the Ru^II^ ground state) is faster in IrO_2_⊂NS‐RuCh. For this latter species, the rate constant of the hole scavenging process is 7.4×10^4^ s^−1^. This is a remarkable value, when compared to the rate constant for the hole scavenging process in the [Ru(bpy)_3_]^2+^/IrO_2_/Na_2_S_2_O_8_ system, which is reported to be 8×10^2^ s^−1^,^[10]^ that is two orders of magnitude slower (this is consistent with the negligible hole scavenging of photogenerated Ru^III^ in NS‐RuCh by “free” IrO_2_ within the measured time window at the IrO_2_ concentrations used; Figure S7). The relevant acceleration of the hole scavenging in IrO_2_⊂NS‐RuCh is attributed to the increase in proximity of both photosensitizer and catalyst subunits, thanks to the restricted environment promoted by the chitosan nanofibers which play the role of concentrators. Such a relevant acceleration of the hole scavenging process is therefore hold responsible for the improvement in the photochemical quantum yield of molecular oxygen production (Table 1), that is in the water oxidation process.

Nanosized fibers IrO_2_⊂NS‐RuCh made of crosslinked Ru‐decorated chitosan have been prepared, with IrO_2_ nanoparticles encapsulated within the fibers by synthetic design. The Ru(bpy)_3_‐type chromophores were covalently linked to the chitosan scaffold. Absorption and emission spectroscopy showed that the Ru^II^ polypyridine units keep their own spectroscopic and photophysical properties essentially unperturbed within the chitosan nanofiber. Photoinduced water oxidation takes place in the system in the presence of persulfate anions as sacrificial agents, with a quantum yield of 0.21, which is significantly higher than the quantum yield obtained for separated Ru^II^ chromophores and IrO_2_ catalyst in solution (0.05), as well as for the Ru‐decorated chitosan nanofibers and non‐encapsulated IrO_2_ nanoparticle catalyst (0.10) investigated for comparison purposes. The more efficient photochemical water oxidation in the IrO_2_⊂NS‐RuCh system is attributed to the restricted environment experienced by the photosensitizers and catalysts, and in particular to kinetic reasons; actually, a remarkably fast hole‐scavenging process between the oxidized photosensitizer and the catalyst occurs in IrO_2_⊂NS‐RuCh, with a rate constant of 7.4×10^4^ s^−1^, which is about two orders of magnitude faster than the same process for isolated light‐harvesting chromophore and catalyst species in solution. This result strongly confirms that restricted environments can play important roles in the design of functional supramolecular assemblies for achieving efficient photochemical water oxidation.

**Supporting Information available**: Details of materials, synthesis and characterization, instrumentation, oxygen evolving experiments.

## Conflict of interest

The authors declare no conflict of interest.

## Supporting information

As a service to our authors and readers, this journal provides supporting information supplied by the authors. Such materials are peer reviewed and may be re‐organized for online delivery, but are not copy‐edited or typeset. Technical support issues arising from supporting information (other than missing files) should be addressed to the authors.

Supporting Information
