# Supplementary material for: Photoinduced Water Oxidation in Chitosan Nanostructures Containing Covalently Linked RuII Chromophores and Encapsulated Iridium Oxide Nanoparticles
Source: Chemistry. 2021 Sep 23;27(68):16904–11. doi: 10.1002/chem.202102032 (PMC9291156; doi:10.1002/chem.202102032)
Supplement: Supplementary file 1 — Supporting Information [file CHEM-27-16904-s001.pdf]

Chemistry–A European Journal

Supporting Information

Photoinduced Water Oxidation in Chitosan Nanostructures Containing Covalently Linked Ru^{II} Chromophores and Encapsulated Iridium Oxide Nanoparticles

Giuseppina La Ganga,* Fausto Puntoriero, Enza Fazio, Mirco Natali, Francesco Nastasi, Antonio Santoro, Maurilio Galletta, and Sebastiano Campagna*

Table of contents

	Page
Materials	2
Synthesis and characterization	2
Instrumentation	3
Oxygen evolving experiments	4
Figure S1	5
Figure S2	6
Figure S3	7
Figure S4	7
Figure S5	8
Figure S6	8
Figure S7	9
References	9

Materials

All reagent used were purchuased from Sigma Aldrich. Chitosan used was Low Molecular Weight, 75-85% deacetylated. Buffer phosphate was prepared by using Romil SPS water.

Synthesis and characterization

bpy-Ch

2,2'-bipyridine-4,4'-dicarboxylic acid (dcbpy) (100 mg, 0,36 mmol) was treated with $SOCl_2$ (5 mL) under reflux condition overnight to give the corresponding acid chloride as a white solid. The product was solubilized in 5 mL of 1,2 dichloroethane and to this one, chitosan (Ch, low molecular weight, 300 mg) and triethylamine (0.5 mL, 3.61 mmol) were added. The mixture was stirred at room temperature for 2 h and then refluxed overnight.[1] The pale pink solid was filtered under vacuum; it was washed several times with CH_2Cl_2 and then diethyl ether.

Table S1. Comparison of founded elemental analysis of **Ch** and **bpy-Ch**

Species	C/%	N/%	H/%	N/C	H/C
**Ch**	53.02	8.24	8.13	15.54	15.33
**bpy-Ch**	54.08	8.51	7.8	15.73	14.42

Elemental analysis shows that coupling of chitosan with 2.2'-bipyridine derivatives has slightly higher N/C ratio and a lower H/C ratio (because bipyridine introduce a subunit with an higher content of nitrogen and lower content of hydrogen atoms with respect to the chitosan monomer).

RuCh

To a suspension of bpy-Ch (100 mg) in ethanol:water 1:1 (12 mL, under N_2 atmosphere) were added 20 mg of $Rubpy_2Cl_2$ (at different time, in 3 different step). The solution was refluxed under N_2 atmosphere for several hours (at least for 4 hours) until the solid suspended changed from pink to orange. The solid was filtered under vacuum; it was washed several times with ethanol and then diethyl ether. From this procedure, it was produced 70 mg of an orange solid (**RuCh**). **^{1}H-NMR** (D_2O, CD_3COOD, 1% v/v); 300 MHz. @ 40°C ;δ, ppm: 9.49 (1 H), 9.05 (1 H), 8.68 (3 H), 8.54-

8,28 (m, 8 H), 8.20 (1 H), 8.04 (d, 2 H), 7.87 (dd,4 H), 7.53(1H), 7.27 (1H), 5.0 (20 H), 4,30 -3,45 (m, 100 H), 3.32 (20 H).

NS-RuCh

An aqueous solution of 9 mL of sodium tripolyphosphate (TPP) (1 mg/ml) was added dropwise to 10 mL in chitosan solution (1mg/ml). The chitosan solution was prepared by dissolving RuCh (35 mg) in a 1% acetic acid aqueous solution. After overnight stirring the RuCh nanostructures (NS-RuCh) were collected by centrifugation at 6000 rpm for 30 min. UV-Vis (phosphate buffer, pH=7),λ_{max}/nm= 465; 287. Emission spectrum (phosphate buffer, pH=7), λ_{emi}/nm= 649.

IrO_2⊂NS-RuCh

An aqueous solution of 9 mL of sodium tripolyphosphate (TPP) (1 mg/ml) was added dropwise to 10 mL in chitosan solution (1mg/ml). The chitosan solution was prepared by dissolving RuCh (35 mg) in a 1% acetic acid aqueous solution and then by adding 1 mL of IrO_2 solution (malonate stabilized, 1.24 mM). [2] After overnight stirring **IrO_2⊂NS-RuCh** were collected by centrifugation at 6000 rpm for 30 min. UV-Vis (phosphate buffer, pH=7),λ_{max}/nm= 650 (sh); 465; 287.

Instrumentation

UV/Vis and luminescence spectra: UV/Vis absorption spectra were taken on a Jasco V-560 spectrophotometer. For steady-state luminescence measurements, a Jobin Yvon-Spex Fluoromax 2 spectrofluorimeter was used, equipped with a Hamamatsu R3896 photomultiplier. The spectra were corrected for photomultiplier response using a program purchased with the fluorimeter.

Luminescence lifetimes: Luminescence lifetimes were determined by time-correlated single-photon-counting Edinburgh OB900 spectrometer (light pulse: 20 Hamamatsu PL2 laser diode, pulse width 59 ps at 408 nm).

SEM-EDX: SEM imaging were carried out using a Zeiss Merlin field emission electron microscope, equipped with a Gemini II column. SEM measurements were performed with an

acceleration voltage of 1.5 kV and at a working distance of 4.5 mm. The SEM apparatus was coupled with a Quanta EDX spectrometer to carry out energy dispersive X-ray (EDX) analysis.

Dynamic Light Scattering analyses: Dynamic Light Scattering (DLS) measurements were performed using the Malvern Instruments Zetasizer NANO-ZS, at 25 ° C.

Laser flash photolysis: Nanosecond transient absorption measurements were performed with an Applied Photophysics laser flash photolysis apparatus, using a frequency-doubled (532 nm, 330 mJ) or tripled (355 nm, 160 mJ) Surelite Continuum II Nd/YAG laser (half-width 6-8 ns) as excitation source. Transient detection was obtained using a photomultiplier-oscilloscope combination (Hamamatsu R928, LeCroy 9360).

Oxygen evolving experiments

The photo-driven oxygen evolving experiments discussed in the main text, have been performed by irradiation ($\lambda > 400$ nm, cut-off filter) of 2 mL of a deoxygenated solution (phosphate buffer, pH 7) containing the photosensitizer ($1x10^{-4}$M), the catalyst ($5x10^{-5}$M) and $Na_2S_2O_8$ (10 mM) in a sealed cell. Oxygen evolution was monitored in a second compartment, following the O_2-dependent emission lifetime of a deoxygenated solution (acetonitrile, 2.5 mL) of $[Ru(bpy)_3]Cl_2$ by a time-correlated single photon counting spectrometer, after quantitative calibration of time-resolved spectral response.

In a photo driven water oxidation system, like the one described in the main text (eqs. 1-5), the efficiency of water oxidation depends on the intensity of the irradiation light and on the absorption of such light by the photosensitizer. For this reason, it is appropriate to compare oxygen production with photons absorbed by the photosensitizer referring to photochemical quantum yield of molecular oxygen produced, $\Phi(O_2)$.

$$\Phi(O_2) = (\text{moles of } O_2 \text{ produced}) / (\text{moles of absorbed photons}) \qquad (6)$$

Moles of photons absorbed were calculated by considering the photon output of irradiation lamp by using Aberchrome 540 as quantum yield standard, according to literature.[3,4]

It has to be noted that $\Phi(O_2)$, calculated by equation 6, can reach a limiting value of 0.25 because four photoinduced cycles are needed to produce the active $C^{(4+)}$ catalyst form, unless radicalic processes are involved. Actually, in the specific case, where persulfate ions are used as the sacrificial agent, the limiting value can reach 0.5, because one of the products of the reaction in eq. 2 (*i.e.* decomposition products) can promptly react with **Ru**, to generate another molecule of oxidized photosensitizer $\mathbf{Ru}^{+}$, or directly contributes to the oxidation of the catalyst, **C**. In this case, two photons are needed for producing a molecule of oxygen.

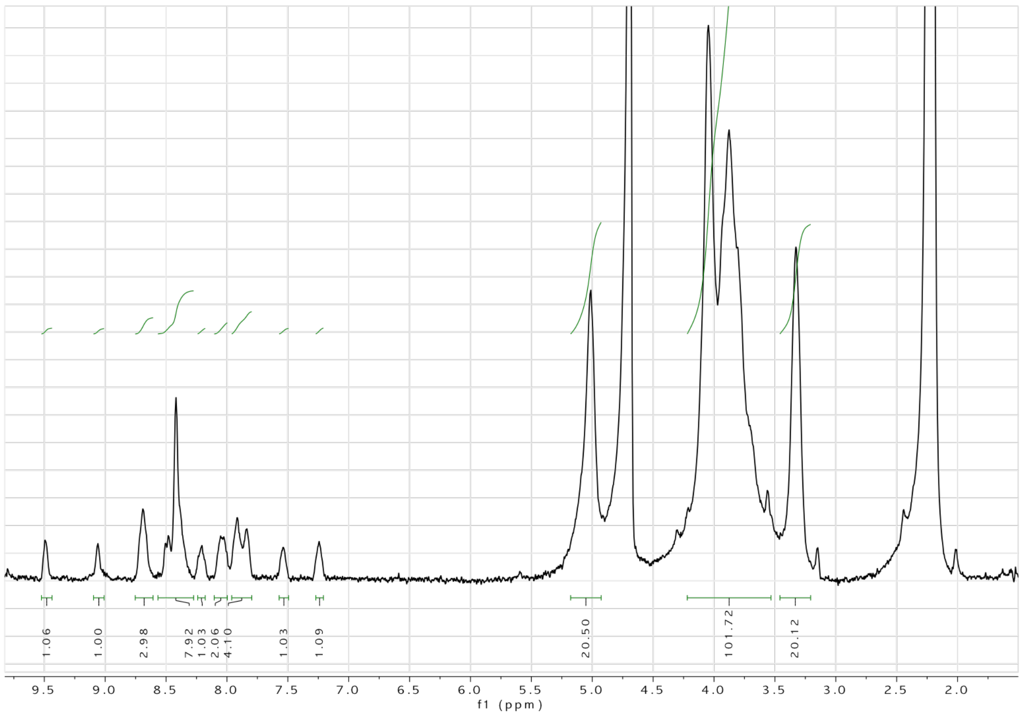

Figure S1 ^{1}H-NMR spectra of **RuCh** in D_2O (CD_3COOD, 1% v/v). Spectra were recorded at 40 °C.

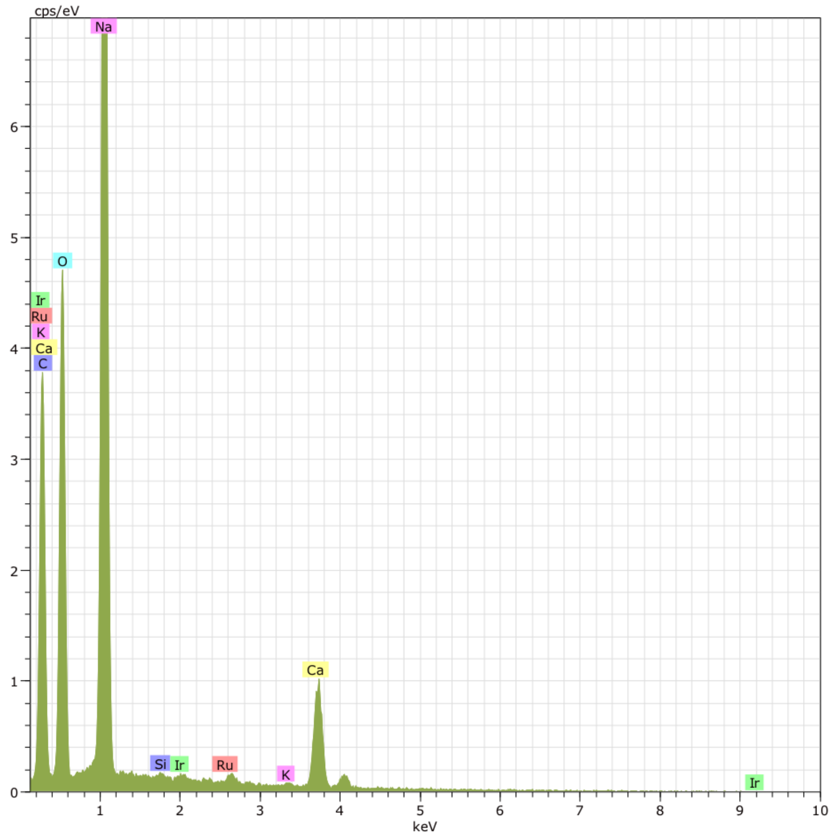

Figure S2 EDX analysis of **IrO_2⊂NS-RuCh.** The species mainly detected are Na, O, Ca, C (synthesis residues and chitosan species with the following percentage [at.%] 22.48%, 35.23%, 5.79%, 36.21%)), Si (the substrate, 0.12%) and Ru and Ir (0.10% and 0.05%). The Ru:Ir ratio is 2:1.

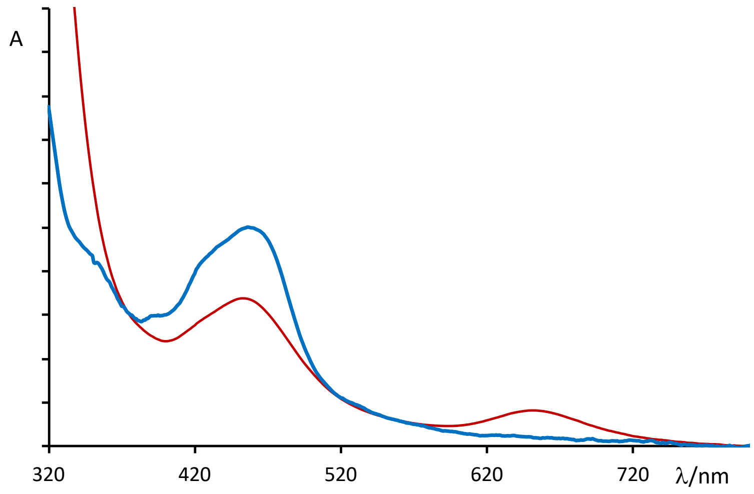

Figure S3. Absorption spectra of **$Ru(bpy)_3Cl_2$ /IrO_2/ $Na_2S_2O_8$** (system **a**) before (blue line) and after (red line) 200 min of photocatalysis.

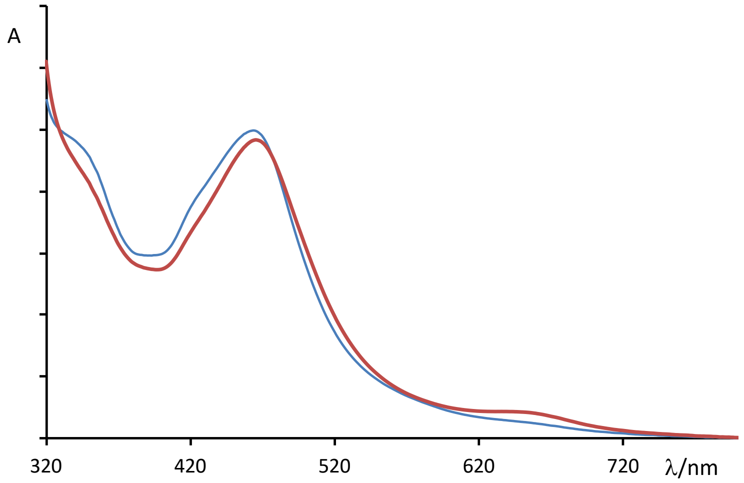

Figure S4. Absorption spectra of **$IrO_2 \subset$NP-RuCh / $Na_2S_2O_8$** (system **c**) before (blue line) and after (red line) 200 min of photocatalysis.

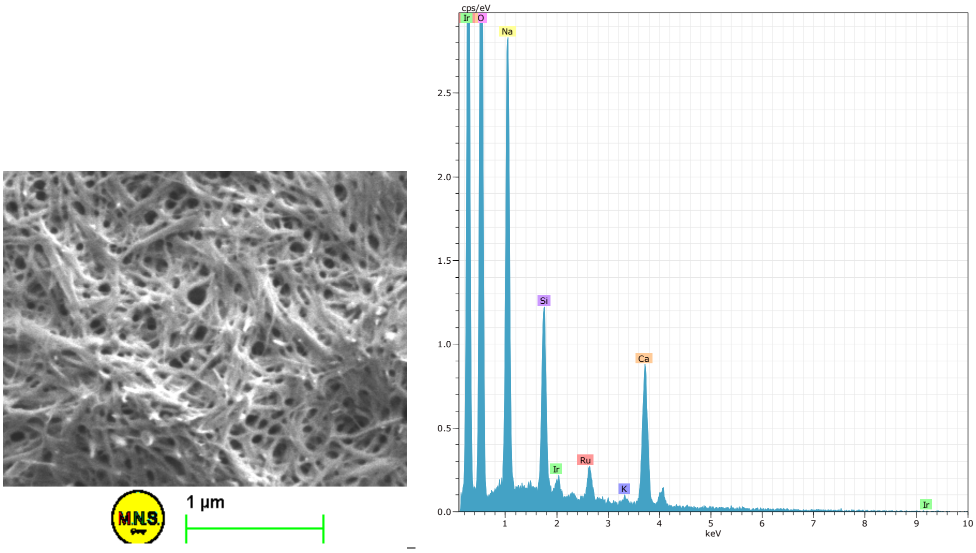

Figure S5. SEM image (left) and EDX analysis (right) of **IrO_2⊂NP-RuCh** (system **c**) after photocatalysis experiments.

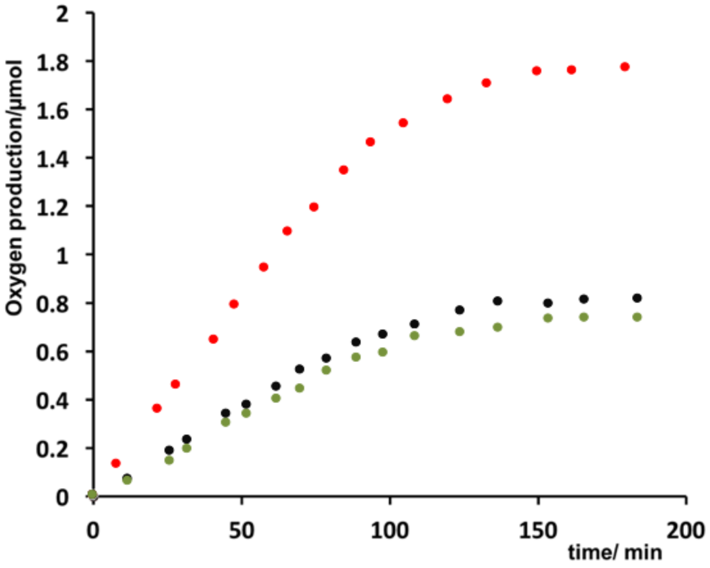

Figure S6. Oxygen evolution in the **IrO_2⊂NP-Ru** / $Na_2S_2O_8$ (red dots), **NP-RuCh** / IrO_2 / $Na_2S_2O_8$ (black dots) and **Ch/Ru(bpy)$_3$Cl$_2$** /IrO_2/ $Na_2S_2O_8$ (green dots) systems.

The **Ch/Ru(bpy)$_3$Cl$_2$ /IrO_2/ $Na_2S_2O_8$** photocatalysis system was obtained adding cross-linked chitosan (20 times more concentrated than Ru(II) photosensitizer), to reproduce conditions used in the other two systems. Photochemical quantum yield for molecular oxygen production: 0.04.

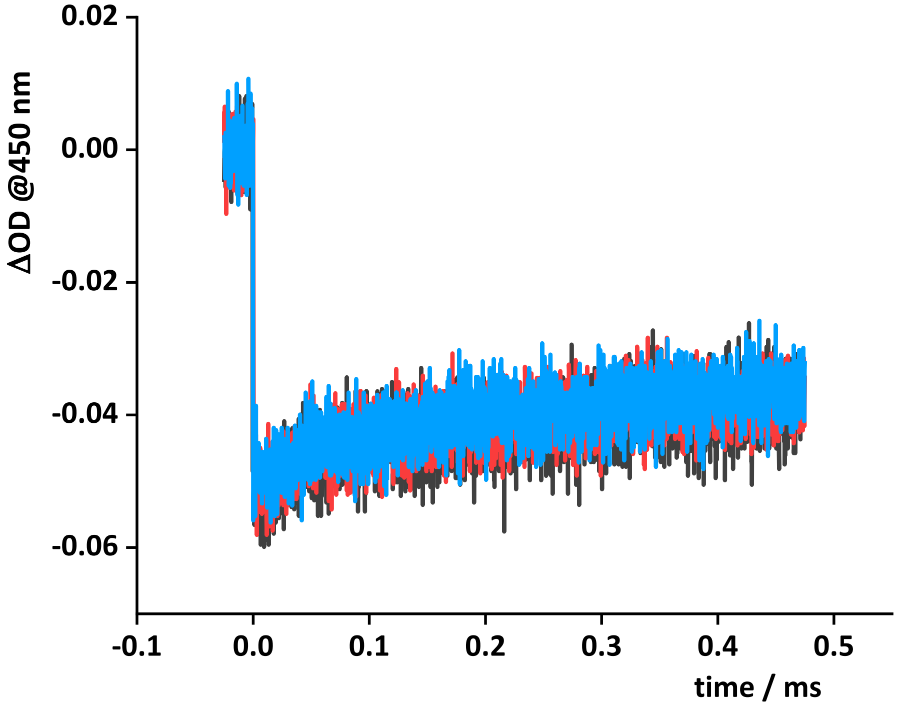

Figure S7 - Flash photolysis experiments: λ_{exc} = 355 nm; $Na_2S_2O_8$ (1 x 10^{-2} M), **NP-RuCh** (black line), **NP-RuCh** / IrO_2 (2.5 x 10^{-5} M, red line), and **NP-RuCh** / IrO_2 (5 x 10^{-5} M, blue line).

References

[1] J. Ohkanda, R. Satoh, N. Kato, *Chem. Commun*. **2009**, 6949–6951.

[2] P. G. Hoertz, Y.-I. Kim, W. J. Youngblood, T. E. Mallouk, *J. Phys. Chem B* **2007**, 111, 6845-6856.

[3] *Handbook of Photochemistry, 3rd Edition* (Eds: M. Montalti, A. Credi, L. Prodi, M. T. Gandolfi), CRC, Boca Raton, **2006**.

[4] (a) G. La Ganga, F. Nastasi, S. Campagna, F. Puntoriero, *Dalton Trans.* **2009**, 9997. (b) G. La Ganga, F. Puntoriero, *Technical note in EPA Newsletter, November **2013**,* pp. 105-112. *ISSN 1011-4246.*
